# Variable wing venation in *Agathiphaga* (Lepidoptera: Agathiphagidae) is key to understanding the evolution of basal moths

**DOI:** 10.1098/rsos.160453

**Published:** 2016-10-05

**Authors:** Sandra R. Schachat, George W. Gibbs

**Affiliations:** 1 Mississippi Entomological Museum, Mississippi State, MS 39762, USA; 2 Department of Paleobiology, Smithsonian Institution, Washington, DC 20013, USA; 3 School of Biological Sciences, Victoria University, PO Box 600, Wellington 6140, New Zealand

**Keywords:** Amphiesmenoptera, development, disparity, morphology, phenotypic variation, polymorphism

## Abstract

Details of the ancestral groundplan of wing venation in moths remain uncertain, despite approximately a century of study. Here, we describe a 3-branched subcostal vein, a 5-branched medial vein and a 2-branched cubitus posterior vein on the forewing of *Agathiphaga vitiensis* Dumbleton 1952 from Vanuatu. Such veins had not previously been described in any Lepidoptera. Because wing veins are typically lost during lepidopteran evolutionary history, rarely—if ever—to be regained, the venation of *A. vitiensis* probably represents the ancestral character state for moths. Wing venation is often used to identify fossil insects as moths, because wing scales are not always preserved; the presence of a supposedly trichopteran 3-branched subcostal vein in crown Lepidoptera may decrease the certainty with which certain amphiesmenopteran fossils from the Mesozoic can be classified. And because plesiomorphic veins can influence the development of lepidopteran wing patterns even if not expressed in the adult wing, the veins described here may determine the location of wing pattern elements in many lepidopteran taxa.

## 1. Introduction

**Figure 1.**
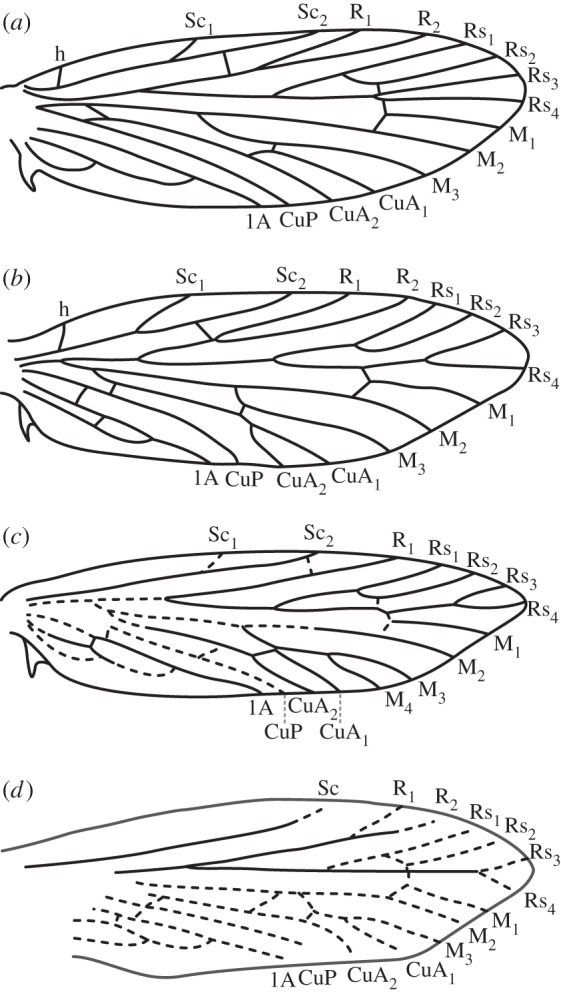
Forewing venation of the most basal crown Lepidoptera. (*a*) A recent reconstruction of the primitive lepidopteran forewing venation groundplan [13]. (*b*) Venation of *Sabatinca calliarcha* Meyrick 1912 [15]. (*c*) Venation of *Agathiphaga queenslandensis* Dumbleton 1952 [16]. (*d*) The only previously published illustration of forewing venation of *A. vitiensis* Dumbleton 1952 [8].

Perhaps because moths have a depauperate fossil record [1] and many early diverging lineages remain undiscovered or undescribed [2], the primitive wing venation groundplan for Lepidoptera is not yet fully understood. Studies of primitive lepidopteran wing venation were common approximately one century ago [3,4], when the three most basal families of Lepidoptera were not yet fully described. Around that time, entomologists debated whether Micropterigidae belonged to the Lepidoptera [5–7]. The discovery and subsequent analysis of Agathiphagidae and Heterobathmiidae [8,9] has revealed that three discrete families of ‘jaw-moths’ comprise the most early diverging families in the order [10–12]; at present, there is no doubt that Micropterigidae, Agathiphagidae and Heterobathmiidae are the three most early diverging families of extant Lepidoptera. But although lepidopteran phylogeny is now very well understood, attempts to reconstruct primitive wing venation are lagging far behind. Recent advances in this area have relied heavily on fossils [13,14] and somewhat unsurprisingly—given the paucity of Mesozoic fossil moths—the latest illustration of primitive lepidopteran wing venation [13] bears a close resemblance to wing venation of Micropterigidae (figure 1*a,b*), the better studied of the two earliest diverging families of extant moths [15].

The phylogenetic position of Agathiphagidae is not entirely understood. Some recent phylogenies have recovered Agathiphagidae the sister group to Micropterigidae, forming a clade that is sister to all other Lepidoptera [10,11,17], but support values are weak and results appear to depend largely on the nature of the dataset that is used; other recent analyses have recovered Micropterigidae alone as sister to all other Lepidoptera [12,18]. Agathiphagidae have informed the primitive groundplan of lepidopteran wing venation in that this family includes the only extant moths with a 4-branched medial (M) vein [16]. Agathiphagidae contains only two described species, both in the genus *Agathiphaga* Dumbleton 1952. In the original description of Agathiphagidae, illustrations of wing venation were incomplete [8]. Since then, illustrations of agathiphagid wing venation have relied on *Agathiphaga queenslandensis* (figure 1*c*), which occurs in mainland Australia [16]. Another agathiphagid species, referred to as ‘*Agathiphaga* sp.,’ was photographed in Australia but has never been collected [19]. The other described agathiphagid species, *Agathiphaga vitiensis*, has rarely been examined since the original, incomplete description (figure 1*d*). *Agathiphaga vitiensis* was first described from Fiji, and additional populations of Agathiphagidae from Vanuatu and the Solomon Islands have also been assigned to this species. Certain aspects of the morphology of *A. vitiensis* have been described [20–23], but since Dumbleton's original description of the family, studies of the wing venation of Agathiphagidae have relied exclusively on *A. queenslandensis* [24]. Notably, the wing venation of *A. queenslandensis* shares multiple features with the venation of the extinct Jurassic family Mesokristenseniidae: a 2-branched subcostal (Sc) vein and a 4-branched M vein [25].

Given the paucity of the lepidopteran fossil record and the lack of attention paid to some of the most early diverging lineages of moths, it is quite possible that recent reconstructions of the primitive lepidopteran wing venation groundplan are incomplete. Because Agathiphagidae are among the most early diverging families of moths, and because they have received so little attention, this family has great potential to further inform reconstructions of primitive lepidopteran morphology.

### 1.1. Wing venation in extinct relatives of Lepidoptera

**Figure 2.**
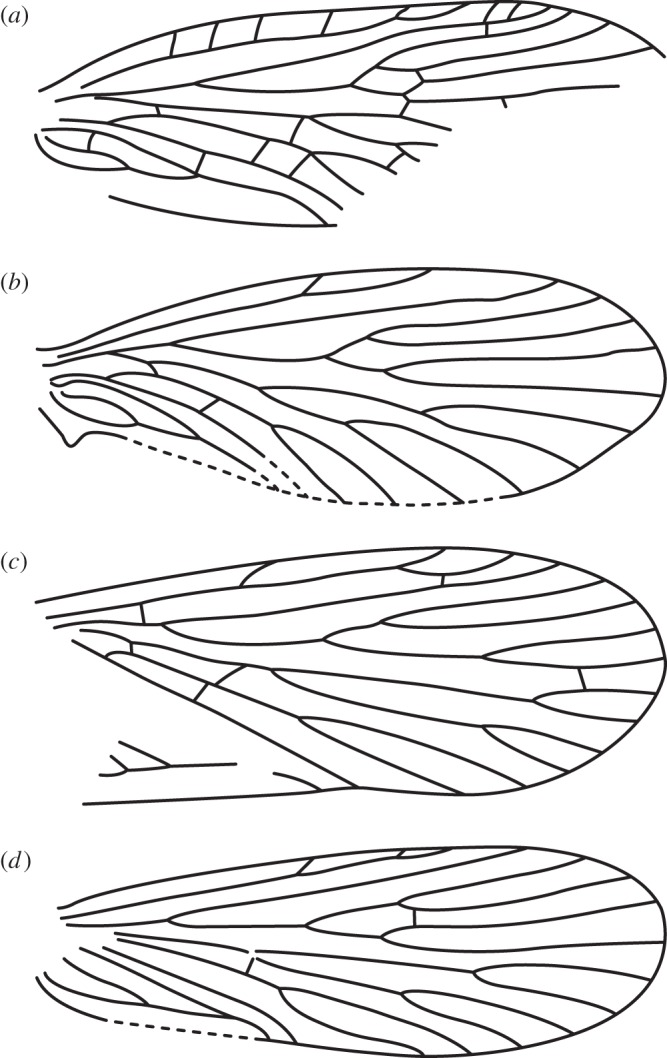
Forewing venation of stem-amphiesmenopteran lineages. (*a*) *Cladochoristella bryani* Riek 1955, which belongs to the earliest known definitive amphiesmenopteran lineage, Cladochoristidae [30,31]. (*b*) *Eocorona iani* Tindale 1980, which belongs to the stem-amphiesmenopteran lineage Eocoronidae [32]. (*c*) The species first described as *Prorhyacophila colliveri* Riek 1955; this species was originally placed in the family Prorhyacophilidae [31], an assignment that was questioned by Ansorge, who synonymized *Prorhyacophila* with *Mesotrichopteridium* [33]. (*d*) *Necrotaulius megapolitanus* Handlirsch 1920 [33], which belongs to the stem-amphiesmenopteran lineage Necrotauliidae.

Before Lepidoptera diverged from stem Amphiesmenoptera, the ancestors of moths had many wing veins. It has been proposed that stem panorpoid insects would have possessed most or all of the wing veins found in extant Mecoptera and Trichoptera [26]; this complete suite of plesiomorphic wing veins is not known in any extant lineage, but can be seen in various Paleozoic fossils [27,28]. The earliest known possible amphiesmenopteran, which dates to the Carboniferous, has many M veins and many subcostal crossveins [29], and during the Permian, Early Panorpoidea including Permotrichoptera had five or more branches of the M vein and three or more subcostal crossveins on the forewing [30]. The earliest definitive Amphiesmenoptera, which belong to the Permo-Triassic family Cladochoristidae [30], often have many subcostal crossveins and four branches of the M vein on the forewing (figure 2*a*).

Families of Amphiesmenoptera that originated during the Mesozoic to recent have fewer subcostal crossveins than the Cladochoristidae [30]; stem Amphiesmenoptera other than Cladochoristidae tend to have two or three (figure 2*b–d*). The earliest definitive fossil lepidopteran, *Archaeolepis mane*, has only one branch of the Sc vein [34,35]. Because early diverging moths in many extant families have a 2-branched Sc vein [16], the fossil record of lepidopteran vein reduction is clearly incomplete, and *Archaeolepis mane* is unlikely to represent the primitive groundplan for the order. The evolution of reduced forewing venation is easier to trace in Trichoptera because early diverging lineages tend to have a more complete suite of plesiomorphic amphiesmenopteran wing veins. For example, certain Trichoptera, such as the extant genus *Rhyacophila*, have long been known to have a 3-branched Sc vein [7,36].

Because a 3-branched Sc vein has not been found in any stem or crown Lepidoptera, one might conclude that a 2-branched Sc vein is the ancestral character state for the order. This is of great importance because wing venation must often be used to classify fossil Amphiesmenoptera; wing scales—an obvious synapomorphy for the Lepidoptera—are often not preserved in fossils. And so, for example, the number of branches of the M vein has been used to assign fossils to Lepidoptera [33]. At present, a fossil amphiesmenopteran with more than two branches of the Sc vein or more than four branches of the M vein would not be considered as possibly belonging to the Lepidoptera.

### 1.2. The predicted location of an ancestral wing vein

**Figure 3.**
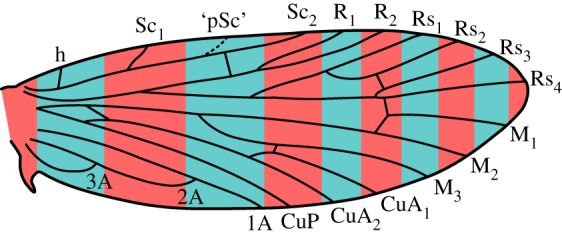
The ‘wing-margin’ model for microlepidopteran wing pattern, consisting of two alternating series of bands in different colours [38,39], with recently updated locations for the branches of the Sc vein [40,41]. The dashed line indicates the location of the predicted plesiomorphic branch of the Sc vein; solid lines indicate wing veins known from Micropterigidae. Either series of bands illustrated here—those in red, or those in blue—could develop a darker colour.

Developmental biologists have known for nearly a century that plesiomorphic veins can continue to influence the development of wing patterning even if not expressed in the adult wing [37]. For microlepidoptera, the only predictive model for wing pattern put forward in recent times [38,39] posits that an ancestral, 3-branched Sc vein continues to influence the development of wing pattern. According to the model, called the ‘wing-margin’ model, the primitive forewing pattern groundplan for Lepidoptera consists of two alternating series of transverse bands, and each transverse band straddles one vein along the costal margin of the wing (figure 3). One more branch of the Sc vein, in addition to the two branches known from many early diverging microlepidoptera, would need to have been present in ancestral moths in order for the relationship between transverse bands and wing veins to be one-to-one along the costa. Studies of Micropterigidae [40,41] narrowed down the location of the plesiomorphic branch of the Sc vein (‘pSc’), positing that it occurs between the visible branches Sc_1_ and Sc_2_ in Micropterigidae (figure 3).

## 2. Material and methods

**Figure 4.**
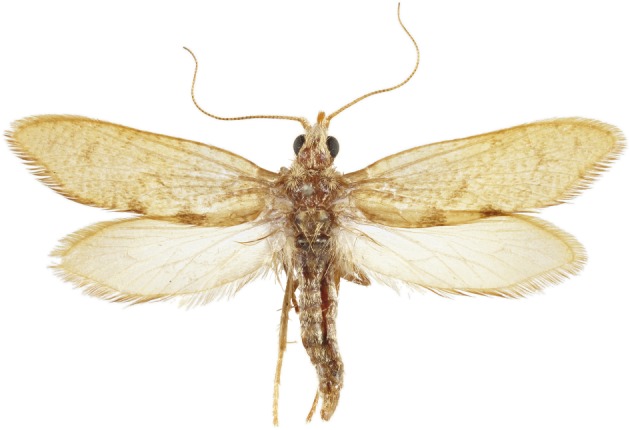
A specimen of *Agathiphaga vitiensis* from Vanuatu held in the Australian National Insect Collection. Photo courtesy of Celia L. Curtis.

Forty-eight spread specimens of *A. vitiensis* from Vanuatu are present in the Australian National Insect Collection in Canberra, Australia (figure 4). Infested seeds of *Agathis microphylla* were collected by A. N. Gillison and P. E. Neil and were transported to Australia, where the adults were reared out by Ian F. B. Common. The adults emerged in 1986. All specimens examined for this study belong to the same population.

Each spread specimen was examined for variation in the number of branches of the Sc and M veins on the forewing. Three specimens were selected for creation of wing slides: one specimen that appeared to have two branches of the Sc vein and four branches of the M vein on the forewing (‘Specimen A’; ANIC 8904 W), one with a 3-branched Sc vein (‘Specimen B’; ANIC 8905 W), and one with a 5-branched M vein (‘Specimen C’; ANIC 8906 W). Though each variant described here—a 2-branched Sc vein; a 3-branched Sc vein; a 4-branched M vein; a 5-branched M vein; a forked cubitus posterior (CuP) vein; and an unforked CuP vein—is illustrated with only one or two specimens, all variants were noted in additional specimens. Total counts for each variant are not provided here because some variants, such as the branching pattern of Sc in Specimen A, are difficult to view on scaled wings. Owing to the scarcity of relevant specimens, a limited number of wing slides were produced and clearings agents (such as Histo-Clear/Histolene) were not used on the remaining specimens.

Wing slides were prepared at Victoria University in Wellington, New Zealand. Photographs were taken at the Australian National Insect Collection on a Leica DSC 500 with the Leica Application Suite software. Final raster images were produced with Affinity Photo software, and vector illustrations were produced with Affinity Designer software.

Terminology is used as follows. Known ancestral veins are said to be ‘lost’ if they do not appear in differentiated form. A vein can appear to be ‘lost’ due to true lack of expression, or due to fusion with an adjacent vein; here, the use of the term ‘lost’ is not intended to imply either mechanism. A vein that is present is said to have been ‘regained’ if it is known to have been present in in a distant ancestor, but absent in a more recent ancestor. ‘Crown’ Lepidoptera is the clade that includes all extant moths and butterflies and their most recent common ancestor; ‘stem’ Lepidoptera are extinct, very early diverging moths that are not descended from the most recent common ancestor of all extant moths and butterflies; and ‘stem’ Amphiesmenoptera are extinct, very early diverging amphiesmenopteran insects that are not descended from the most recent common ancestor of all extant caddisflies, moths and butterflies.

## 3. Results

**Figure 5.**
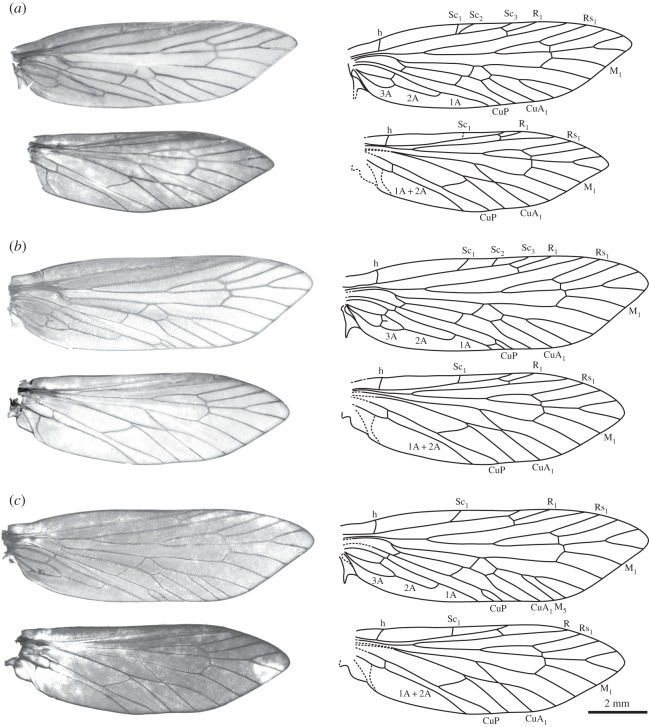
Wing venation in *Agathiphaga vitiensis* from Vanuatu; all specimens are from the same population. (*a*) Specimen A (ANIC 8904W) has four branches of the M vein and an unbranched CuP vein; the second branch of its Sc vein is very close to the first. (*b*) Specimen B (ANIC 8905W) has three evenly spaced branches of the Sc vein and a branched CuP vein with a crossvein connecting CuP_2_ to 1A. (*c*) Specimen C (ANIC 8906W) has five branches of the M vein and a branched CuP vein with no crossvein connecting CuP_2_ to 1A. All images are to the same scale.

**Figure 6.**
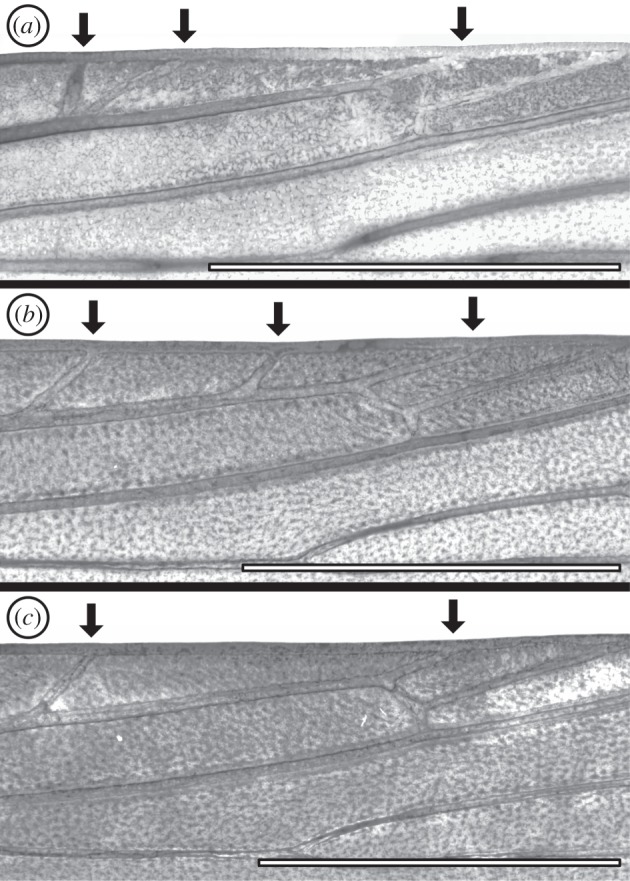
Details showing Sc veins on the forewing. Specimens (*a*) (ANIC 8904W), (*b*) (ANIC 8905W) and (*c*) (ANIC 8906W). An arrow indicates each branch of the Sc vein. Scale bars represent 2 mm.

*Agathiphaga vitiensis* from Vanuatu has variable forewing venation, showing a high degree of intraspecific polymorphism. A wing slide was made from Specimen A (ANIC 8904 W), because this specimen initially appeared to have the same forewing venation as *A. queenslandensis* (figure 1*c*): two branches of Sc, four branches of M and an unbranched CuP. However, once the wing slide was prepared, it became clear that Specimen A has a 3-branched Sc vein, with the second branch occurring very close to the first (figures 5*a* and 6*a*). Specimen B (ANIC 8905 W) has a 4-branched M vein (figure 5*b*), just like *A. queenslandensis*, but it also has a 2-branched CuP vein and a 3-branched Sc vein (figure 6*b*). In this specimen, CuP_2_ is connected to 1A by a crossvein. Specimen C (ANIC 8906 W) has a 5-branched M vein and a 2-branched CuP vein, though here, CuP_2_ is not connected to 1A by a crossvein (figure 5*c*); of the three specimens chosen for the preparation of wing slides, only Specimen C has a 2-branched Sc vein (figure 6*c*). The branch of Sc that is present in some, but not all, specimens occurs in the same location that was predicted by the ‘wing-margin’ model.

In summary, variation in forewing venation illustrated here includes: 2-branched and 3-branched Sc veins, with variable positioning of Sc_2_; 4-branched and 5-branched M veins; and unbranched and branched CuP veins, with and without CuP_2_–1A crossveins. Other notable variants of forewing venation not illustrated here include an unbranched Sc vein and an unbranched R vein.

Comparison of the Sc vein on the forewing of different specimens shows that, when only two branches are visible (figure 6*c*), these correspond to Sc_1_ and Sc_3_ in specimens where all three branches are present (figure 6*a*,*b*). Because Sc_2_ is the branch of Sc that is least frequently expressed in *A. vitiensis*, it is probably also this branch that is not expressed on the adult wing of other Lepidoptera. Therefore, the branch of Sc known only from *Agathiphaga* occurs precisely in the location predicted by studies of microlepidopteran wing pattern (figure 3).

The hindwings of the specimens examined here are generally consistent with previous expectations. The hindwings have an unbranched or 2-branched Sc vein, a 3-branched M vein and an unbranched CuP vein (figure 5). The hindwings appear to have a basal lobe, which was included in the vector illustrations. However, unlike the jugum, the basal lobe is a remnant of an axillary sclerite and therefore should not be considered as a part of the membranous wing.

## 4. Discussion

**Figure 7.**
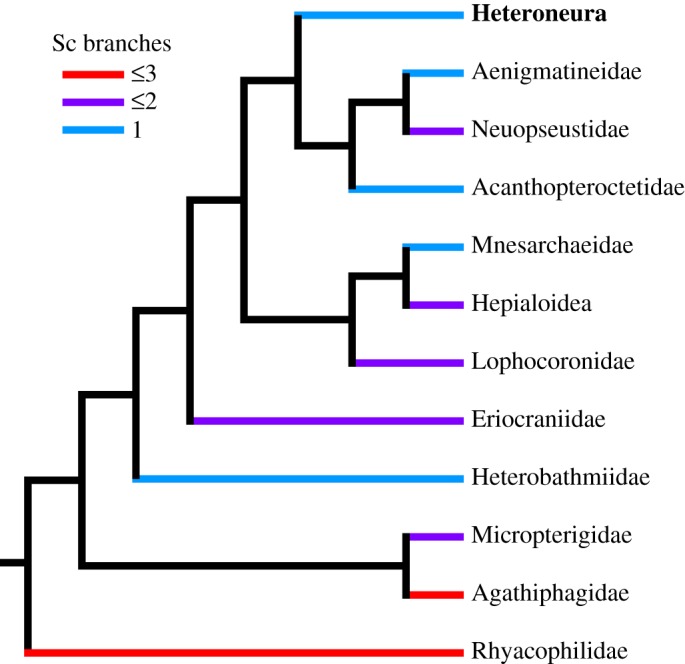
The number of branches of Sc vein in homoneurous moths, plotted onto a recent phylogeny [17]. The trichopteran family Rhyacophilidae, used as an outgroup in molecular studies of early diverging moths [15], is included here as the sister group to Lepidoptera.

**Figure 8.**
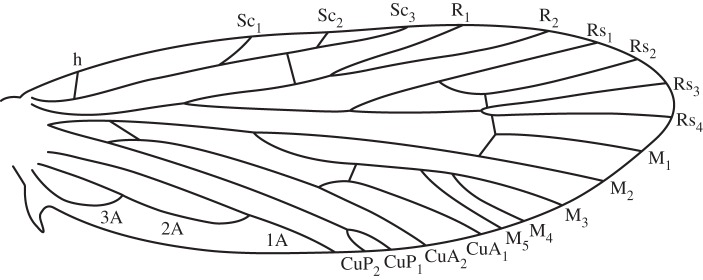
A new hypothesis for the primitive forewing venation groundplan for Lepidoptera. This is an updated version of previous groundplans [13,51], with modifications based on the findings reported here.

Published descriptions of forewing venation in homoneurous moths [9,11,16,24,42–45] show that 2-branched Sc veins are most common in the most early diverging families, such as Micropterigidae, Eriocraniidae and Lophocoronidae. With the exception of just a single species [46], heteroneurous moths have an unbranched Sc vein on the forewing [47]. When the number of Sc branches is plotted onto a phylogeny (figure 7), it is not clear from the phylogeny alone whether a 2-branched Sc vein has been lost and then regained in certain families such as Neopseustidae, or whether parallelisms in the number of branches of Sc vein are due to independent loss of the second branch in families such as Heterobathmiidae and Mnesarchaeidae. However, because there is only one possible example of a 3-branched Sc vein ever regained in any Lepidoptera, and because a 2-branched Sc vein is known only from the most early diverging families, the general trend is that branches of the Sc vein have been lost during evolutionary history, rarely to be regained. Probable cases of lost veins being regained are known from Gelechioidea [48], particularly Elachistidae [49,50]—subject to further resolution of the phylogenetic topology—and a multi-branched Sc vein is known to have been regained in a single tineoid species, *Arrhenophanes perspicilla* [46]. However, these scattered examples among Heteroneura suggest that veins are very infrequently regained. Therefore, at present, the most plausible conclusion regarding the 3-branched Sc vein, 5-branched M vein and 2-branched CuP vein on the forewing of *A. vitiensis* is that these represent the primitive character states for crown Lepidoptera (figure 8).

As stated above, veins can appear to be ‘lost’ due to either true lack of expression, or fusion with an adjacent vein. Intraspecific variation in wing venation is often attributed to fusion (e.g. [52]). Because 4- and 5-branched M veins and 2-branched CuP veins are not known from any Lepidoptera other than *Agathiphaga*, it is unclear whether the extra branches of these veins truly are not expressed in other taxa, or have fused with adjacent branches. But because a 4-branched Sc vein is known from the tineoid species *Arrhenophanes perspicilla* [46], the most probably explanation of loss of Sc branches in Lepidoptera is fusion. It is possible that the first branch of Sc in *Arrhenophanes perspicilla* is actually the humeral vein; otherwise, the first instance of fusion, which would have reduced the number of branches of Sc from four to three, may have occurred before crown Lepidoptera diverged from stem Amphiesmenoptera. The morphology of *A. vitiensis* suggests that additional instances of fusion would have occurred within crown Lepidoptera.

Recent large-scale phylogenies of Lepidoptera have recovered varying topologies for Micropterigidae, Agathiphagidae, Heterobathmiidae and Glossata, often characterized by low support values [10–12,17]. Because the lepidopteran fossil record is so depauperate, the relative timing of the divergences between these lineages is unknown; since ancient, rapid divergences are notoriously difficult to resolve [53], a well-supported phylogeny for Micropterigidae, Agathiphagidae, Heterobathmiidae and Glossata may be difficult to achieve. Regardless, Agathiphagidae are undoubtedly among the most early diverging Lepidoptera.

According to ‘Rosa's rule of “progressive reduction of variability,”’ intraspecific variability is greatest in early diverging lineages [54]. Some palaeontological studies have found early diverging lineages to be characterized by relatively high morphological variability. For example, polymorphisms are more common among basal trilobite species [55]. Fossil data are needed to evaluate Rosa's rule [56,57] and are not available for *Agathiphaga*—it is unclear whether the heightened frequency of intraspecific polymorphism reported here is an ancestral or derived condition.

### 4.1. Potential future studies

A variety of further studies should be carried out in order to test this new reconstruction of primitive lepidopteran wing venation. It is not clear how many *A. queenslandensis* specimens have been examined for variation in wing venation; the venation of *A. vitiensis* from Fiji appears not to have been examined since Dumbleton's original, incomplete description (figure 1*d*), and the venation of *A. vitiensis* from the Solomon Islands appears never to have been examined. Examination of *A. vitiensis* specimens from Vanuatu that are held in other collections may reveal more variants, and should provide a better idea of the relative frequencies of the variants reported here.

In his original description of *Agathiphaga*, Dumbleton predicted that the genus may occur on other islands where its plant host, *Agathis*, is present [8]. The 17 living species of *Agathis* are distributed throughout the southern Pacific in Borneo, Malaysia, New Caledonia, New Guinea, New Zealand and Sumatra in addition to Fiji, Vanuatu, the Solomon Islands and Australia [58]. Because the homoneurous moths of New Caledonia and New Zealand are already very well studied [15,59], it is not particularly likely that new species of *Agathiphaga* await discovery in either of these regions. However, it is certainly possible that *Agathiphaga* occurs in various other areas of the Pacific.

*Agathis* fossils are known from the Cenozoic of Australia [60], New Zealand [61] and Patagonia [62], but no Agathiphagidae have been reported in association with the fossil plants. There are no reported fossil occurrences of *Agathis* or Agathiphagidae from the Mesozoic. A recent analysis found that crown *Agathis* originated during the Cenozoic, and diverged from its sister genus *Wollemia* either during the Cretaceous or Paleogene [63]. Before *Agathis* originated, the ancestors of *Agathiphaga* may have been associated with stem Araucariaceae, which had originated by the Early Triassic [63]. Cenozoic deposits that contain *Agathis* and Mesozoic deposits that contain Araucariaceae are probably the most promising locations to search for fossil Agathiphagidae. Future studies of extant *Agathiphaga* and of extinct moths have great potential to provide broader context for the results reported here.

## 5. Conclusion

The forewings of *A. vitiensis* specimens from Vanuatu contain three veins that had not been previously described in any moths: a 3-branched Sc vein, a 5-branched M vein and a 2-branched CuP vein. The third branch of Sc occurs exactly where it was predicted to occur based on previous studies of the relationship between wing pattern and wing venation [40,41]. Because the existence and location of this vein were predicted based on wing pattern alone, the presence of this vein in the ancestral lepidopteran groundplan should be taken into consideration during future studies of the evolution and development of wing pattern in moths and butterflies. The high variability of vein branching patterns among these basal moths suggests that fossil moths should not be assigned to separate species based only on differences in wing venation, and that great caution should be used when classifying amphiesmenopteran fossils based on venation alone.

## Data accessibility

This study is based on observations of wing slides, all of which are figured in the manuscript.
